# Supplementary material for: Dream Recall upon Awakening from Non-Rapid Eye Movement Sleep in Older Adults: Electrophysiological Pattern and Qualitative Features
Source: Brain Sci. 2020 Jun 3;10(6):343. doi: 10.3390/brainsci10060343 (PMC7349242; doi:10.3390/brainsci10060343)
Supplement: Supplementary file 1 [file brainsci-10-00343-s001.zip › brainsci-827350-supplementary/Supplementary Materials.pdf]

Supplementary Materials

To ascertain that no difference was present in other EEG bands (theta, alpha and sigma), we carried out supplementary analyses. No statistically significant difference was found between groups.

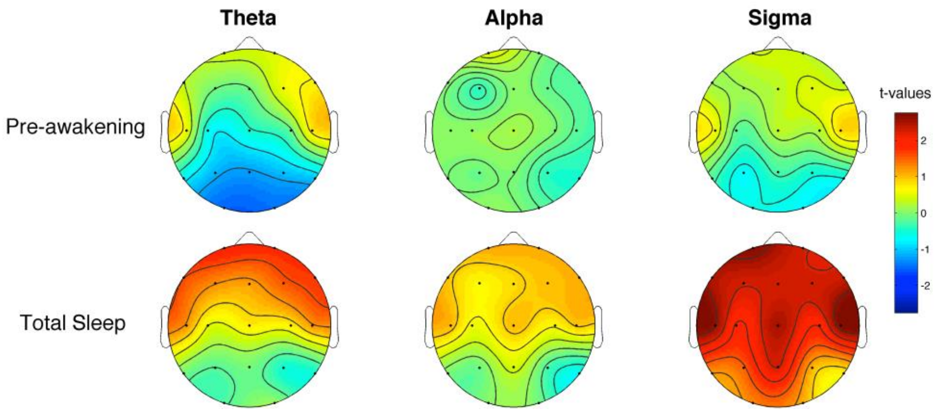

Figure S1. Statistical maps of the comparisons between REC and NREC group on theta, alpha and sigma bands. Statistical maps of the comparisons (unpaired t-test) between NREC and REC group are plotted for theta, alpha and sigma band, both for the pre-awakening (1st row) and the total sleep (2nd row). The statistical maps are scaled symmetrically according to the absolute maximal t-value across the statistical comparisons.

For illustrative purposes, here, we show the topographic distribution of the Pearson's r coefficients of the correlations between the delta activity (during the entire night) and the visual vividness (VV). The figure reveals that both right and left centro-temporo-parietal areas report a negative correlation between the delta activity and VV.

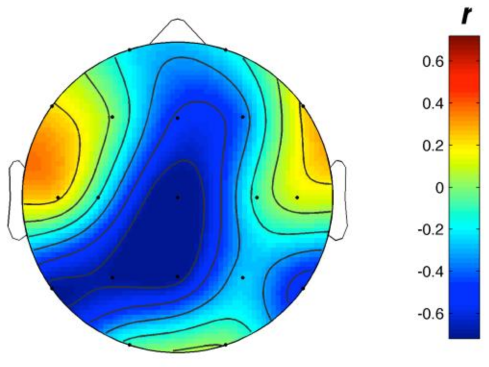

Figure S2: Topographic distribution of the Pearson's *r* coefficients of the correlations between the delta power and the VV. Values are expressed in terms of r values: positive values indicate the presence of a positive correlation, and viceversa. Values are colour coded, plotted at the corresponding position on the planar projection of the hemispheric scalp model and interpolated between electrodes.

For illustrative purposes, here, we show the topographic distribution of the Pearson's *r* coefficients of the correlations between the activation index values (during the entire night) and the self-reported length (sr_L) of dream reports. The figure reveals a negative correlation between the two variables spatially diffuse over the scalp with a peak in the centro-parietal area.

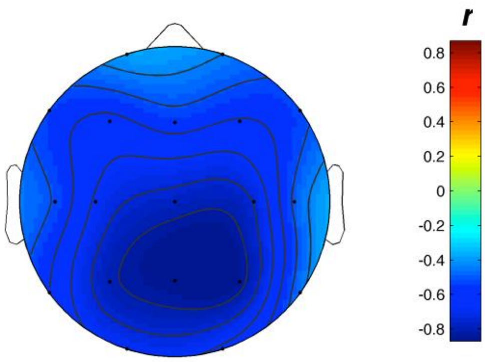

Figure S3: Topographic distribution of the Pearson's r coefficients of the correlations between the activation index values and the sf_L of dream report. Values are expressed in terms of r values: positive values indicate the presence of a positive correlation, and viceversa. Values are colour coded, plotted at the corresponding position on the planar projection of the hemispheric scalp model and interpolated between electrodes.
